# Shigella-specific antibodies in the first year of life among Zambian infants: A longitudinal cohort study

**DOI:** 10.1371/journal.pone.0252222

**Published:** 2021-05-27

**Authors:** Caroline C. Chisenga, Samuel Bosomprah, Michelo Simuyandi, Katayi Mwila-Kazimbaya, Obvious N. Chilyabanyama, Natasha M. Laban, Anya Bialik, Valeria Asato, Shiri Meron-Sudai, Gad Frankel, Daniel Cohen, Roma Chilengi

**Affiliations:** 1 Centre for Infectious Disease Research in Zambia, Lusaka, Zambia; 2 Department of Biostatistics, School of Public Health, University of Ghana, Accra, Ghana; 3 School of Public Health, Sackler Faculty of Medicine, Tel Aviv University, Tel Aviv, Israel; 4 Imperial College London, London, United Kingdom; Public Health England, UNITED KINGDOM

## Abstract

**Introduction:**

Shigellosis, is a leading cause of moderate-to-severe diarrhoea and related mortality in young children in low and middle income countries (LMICs). Knowledge on naturally acquired immunity can support the development of *Shigella* candidate vaccines mostly needed in LMICs. We aimed to quantify *Shigella-*specific antibodies of maternal origin and those naturally acquired in Zambian infants.

**Methods:**

Plasma samples collected from infants at age 6, 14 and 52-weeks were tested for *Shigella* (*S*. *sonnei* and *S*. *flexneri* 2a) lipopolysaccharide (LPS) antigen specific immunoglobulin G (IgG) and A (IgA) by enzyme-linked immunosorbent assay.

**Results:**

At 6 weeks infant age, the IgG geometric mean titres (GMT) against *S*. *sonnei* (N = 159) and *S*. *flexneri* 2a (N = 135) LPS were 311 (95% CI 259–372) and 446 (95% CI 343–580) respectively. By 14 weeks, a decline in IgG GMT was observed for both *S*. *sonnei* to 104 (95% CI 88–124), and *S*. *flexneri* 2a to 183 (95% CI 147–230). Both *S*. *sonnei* and *S*. *flexneri* 2a specific IgG GMT continued to decrease by 52 weeks infant age when compared to 6 weeks. In 27% and 8% of infants a significant rise in titre (4 fold and greater) against *S*. *flexneri* 2a and *S*. *sonnei* LPS, respectively, was detected between the ages of 14 and 52 weeks.

IgA levels against both species LPS were very low at 6 and 14 weeks and raised significantly against *S*. *flexneri* 2a and *S*. *sonnei* LPS in 29% and 10% of the infants, respectively.

**Conclusion:**

In our setting, transplacental IgG anti-*Shigella* LPS is present at high levels in early infancy, and begins to decrease by age 14 weeks. Our results are consistent with early exposure to Shigella and indicate naturally acquired IgG and IgA antibodies to *S*. *flexneri* 2a and *S*. *sonnei* LPS in part of infants between 14 and 52 weeks of age. These results suggest that a potential timing of vaccination would be after 14 and before 52 weeks of age to ensure early infant protection against shigellosis.

## Introduction

Generally, by the age of five, infants suffer repeated bouts of diarrhoea [1] and account for approximately 688 million illnesses and 499,000 deaths [2, 3]. Majority of these deaths (90%) occur in South Asia and sub-Saharan Africa [4]. The Global Enteric Multicentre Study (GEMS study) on burden and aetiology of moderate-to-severe (MSD) diarrhoeal disease in children aged <5 years found that *Shigella* was among the four most common pathogens across sites and age strata in Africa and Asia [5]. In Zambia, *Shigella* is reported to be the second leading attributable cause of MSD in children <5 years old with an estimated prevalence of 35% [6]. There are four *Shigella* serogroups, *Shigella flexneri* (*S*. *flexneri)*, *Shigella sonnei* (*S*. *sonnei)*, *Shigella dysenteriae* (*S*. *dysenteriae*) and *Shigella boydii* (*S*. *boydii*) with >40 identified serotypes across these groups. Previous reports show that in low- and middle-income countries (LMICs), *S*. *flexneri* 2a accounts for most cases of shigellosis in children <5 years old [5] while in high income countries *S*. *sonnei* is said to be responsible for the vast majority of cases [7]. However, there has been a notable shift towards increasing rates of *S*. *sonnei* infections being reported in LMICs now than before [8].

The burden of disease and the emerging global increase in antimicrobial resistance of *Shigella* [9, 10] clearly demand for enhanced interventions to avert the reported cases and deaths. The primary prevention of Shigellosis is based on universal access to safe water, improved sanitation and personal and food hygiene [11]. Development of an efficacious and affordable vaccine would complement and accelerate disease reduction, particularly in LMICs where primary preventive methods are practically unattainable in the short to medium term. Unfortunately, there is a dearth of both epidemiological and immunological information on *Shigella* infection and disease from endemic settings, a gap which needs to be filled to support important vaccine development and deployment work.

*Shigella* infection confers around 70% serotype specific immunity for a limited period of time [12, 13]. Data from both high and middle income countries (HMICs) [14] and LMICs [15] showed that the incidence of culture proven Shigellosis is lowest in the first year of life in children <5 years old and it is postulated that this is due to protective levels of *Shigella* specific serum immunoglobulin G (IgG) of maternal origin [16]. Presence of *S*. *sonnei* and *S*. *flexneri* 2a anti-lipopolysaccharide (LPS) IgG in cord blood and it’s strong correlation with levels in mothers colostrum immediately after delivery has been reported [17]. In Vietnamese infants, the median half-life of maternal anti-*S*. *sonnei* LPS IgG was 43 days after birth [18]. Following a finding that *Shigella* specific anti-LPS IgG antibodies were strongly associated with protection in sero-epidemiological studies carried out in Israel [19, 20], injectable *Shigella* conjugate vaccines have been developed [21]. The *S*. *sonnei* conjugate vaccines showed 74% protective efficacy in young adults and 71% protection in children aged 3–4 years in efficacy trials in Israel, but failed to protect children less than 3 months old [22]. The studies also showed that serum IgG anti-LPS levels correlated with protection.

Recently, second generation and new formulations of monovalent subunit parenteral *Shigella* vaccine candidates including synthetic carbohydrate-based conjugates [23], bio conjugates [24] as well as the Generalized Modules for Membrane Antigen (GMMA) particles have been developed [25]. The *S*. *sonnei* vaccine (1790GAHB) also showed good immunogenicity in phase 2a study with significant increases in geometric mean concentration between baseline and 29 days after a single dose [26]. Also some preliminary protective efficacy in controlled human infection challenge studies in adults in the US where also observed with Flexyn2a, a candidate bioconjugate vaccine against *S*. *flexneri 2a* [27]. While additional challenge studies are presently underway, the next important stage ought to be the evaluation of the immunogenicity and safety of these candidates in paediatric age descending studies in LMICs.

Therefore, we sought to quantify serum antibodies specific to *S*. *flexneri* 2a and *S*. *sonnei* LPS in Zambian infants and assess duration of specific IgG of maternal origin. We aimed to also propose a potential window for future infant immunisation with a *Shigella* injectable vaccine in relation to the timing of early natural exposure to *Shigella* as documented by IgG and IgA seroconversion to *Shigella* LPS.

## Methods

### Study design and participants

We used existing data and stored biological samples collected from a longitudinal cohort of mother-infant pairs previously enrolled in an observational rotavirus vaccine study at Kamwala Clinic in Lusaka Zambia. Kamwala Clinic is a peri-urban Primary Healthcare Facility providing basic outpatient and maternal child health services to a low-income catchment population.

Briefly, participants were enrolled during their routine immunisation clinic visits. The study staff approached the mother-infant pairs at the initial visit with study material. Interested individuals were provided with detailed study information and consented accordingly. A comprehension test was administered and only those meeting the minimum understanding provided individual written consent. Illiterate mothers were provided information through an impartial witness who provided a signed confirmation together with thumbprint of the participant as confirmed written consent. Principally, the pair was eligible if: i) there was willingness by the mother to participate including provision of a signed informed consent; ii) the infant was eligible for rotavirus vaccine immunisation per national policy (male or female infant, aged 6–12 weeks old); iii) the mother was willing and available to undergo study procedures such as questionnaires, HIV counselling and testing, CD4 testing, and provide breastmilk at enrolment; iv) the mother was ready to allow her infant to also receive full-course rotavirus vaccination, phlebotomy at enrolment and 1 month post full rotavirus vaccination, and presentation to clinic for collection of stool sample when infant had diarrhoea; and v) intended to remain in their locality and was ready to attend all scheduled visits for the duration of the study [28]. All data was completely de-identified before we accessed it. Although the parent study was granted approval to conduct HIV testing on the mothers, approval for HIV testing in the infants was not obtained due to insufficient sample and therefore HIV status could not be determined.

### Sample profile

**Fig 1:**
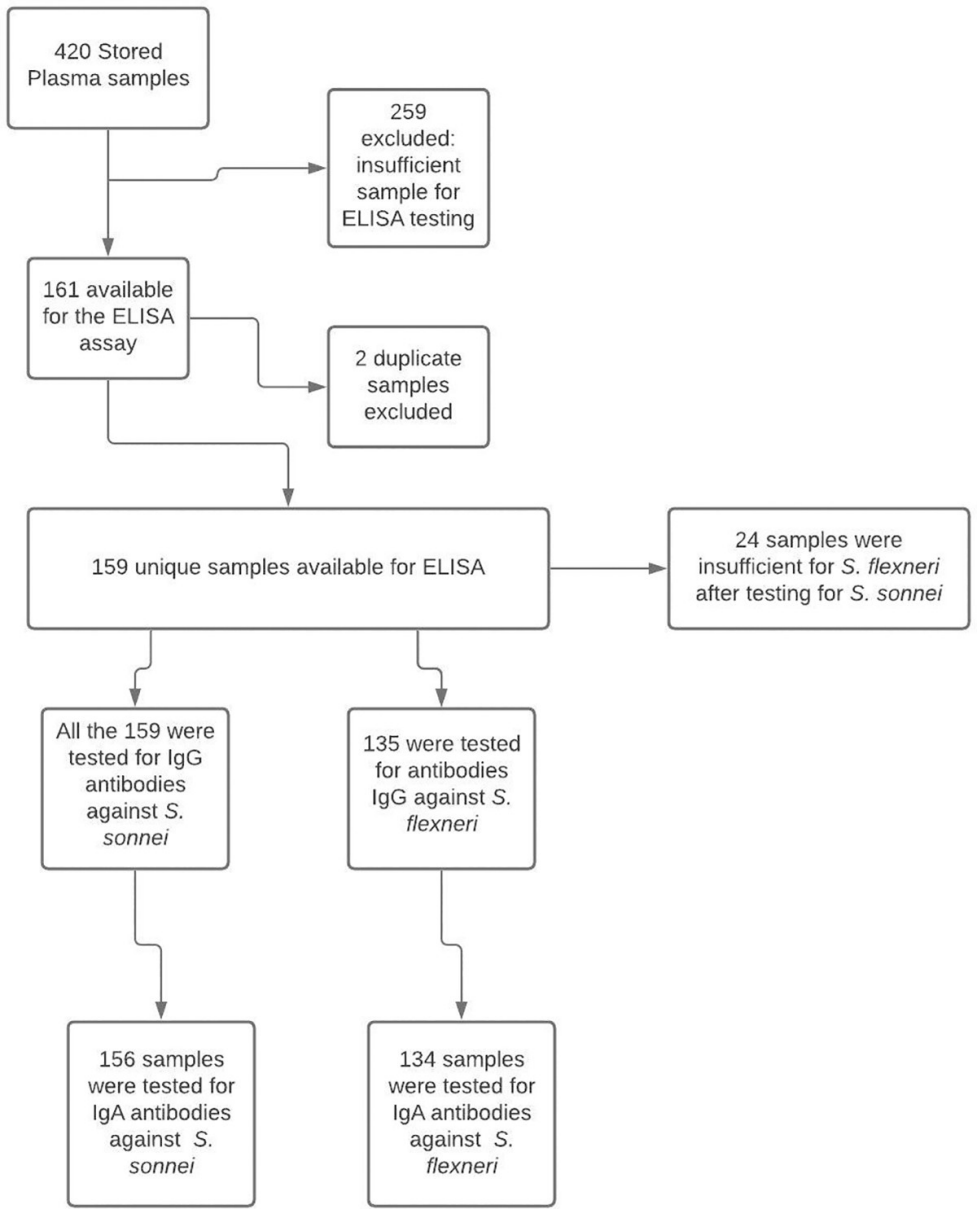
Sample flow diagram.

The original study enrolled 420 mother-infant pairs attending routine infant immunisation clinics between April 2013 and March 2014, and who were followed until mid-2017. Plasma samples were collected from the infants at ages 6, 14 and 52 weeks and stored at -80°C. In the current study, we used the left over plasma samples to test for *Shigella* specific antibodies in Zambian infants. From a total of 420 leftover serum samples, 259 were insufficient and excluded accordingly. Of the remaining 161, 2 duplicate samples were deleted giving us 159 Fig 1.

A total of 159 longitudinal plasma samples collected from infants at ages 6, 14 and 52 weeks were tested for IgG antibodies to *S*. *flexneri* 2a LPS and 135 were tested for IgG antibodies *S*. *sonnei* LPS. IgA antibodies to the same antigens (134 triplets of sera to *S*. *flexneri* 2a LPS and 156 to *S*. *sonnei* LPS) were also tested. Socio-demographic, clinical and anthropometric data for both infants and their mothers were obtained from the existing database of the rotavirus vaccine study.

### Laboratory procedures

#### Shigella specific enzyme-linked immunosorbent assay

Enzyme-Linked Immunosorbent Assay (ELISA) for *S*. *flexneri* 2a and *S*. *sonnei* specific LPS IgG and IgA antibodies was conducted at the Tel Aviv University School of Public Health and was performed as previously described [29]. Briefly, 96-well polystyrene microtiter plates (cat. No. 3590; Corning-Costar) were coated with 5μg/ml of *S*. *flexneri* 2a LPS or 0.5μg/ml of *S*. *sonnei* LPS in 0.05 M carbonate buffer (pH 9.6) for 1 hour at 37°C. After removal of coating solution, unbound sites were blocked through 1-hour incubation at 37°C. The wells were then washed twice with phosphate buffered saline containing 0.05% Tween 20 (PBST). Duplicates of test plasma samples 2-fold serial diluted (8 dilutions) in blocking buffer were added to coated wells and incubated overnight at room temperature. The plates were then washed four times with PBST and alkaline phosphatase conjugated antibody anti-human IgG (cat. No. 5220–0348, KPL antibodies and conjugates; Sera Care, MA, USA) or IgA (cat. No.5220-0347, KPL antibodies and conjugates; Sera Care, MA, USA) diluted at 1:5000 and 1:8000, respectively, were then added and incubated overnight at room temperature. Wells were washed four times with PBST and phosphatase substrate (para-Nitrophenylphosphate-pNPP One component Microwell Substrate Solution, cat. No. 0421–01, Southern Biotech, AL, USA) was added for 15 min at room temperature. Colour development was stopped by the addition of 3M Sodium Hydroxide. Optical density (OD) was measured at 405nm using an ELISA plate reader (Thermo Scientific *Multiskan FC*; MA, USA). OD values were corrected by subtraction of OD value of blank wells. Results were expressed in end point titres (the last serum dilution yielding an OD of 0.2 or higher).

### Statistical analysis

Social demographic characteristics of the participants were summarised using frequencies. We computed geometric mean titres (GMT) and 95% CI for both *S*. *sonnei* and *S*. *flexneri 2a* IgG and IgA titres at 6 weeks, 14 weeks and 52 weeks. Seroconversion for both *S*. *sonnei* and *S*. *flexneri 2a* was defined as at least a 4-fold rise in IgG between 6 weeks and 14 weeks and between 14 weeks and 52 weeks. We compared differences in seroconversion by demographic characteristics using Pearson chi-square test and Fisher’s exact test as appropriate. A p-value of 0.05 or less was considered to be statistically significant. All statistical analyses were performed in Stata 16 MP2 (StataCorp, College Station, TX, USA).

### Ethical approval

The University of Zambia Biomedical Research Ethics Committee approved the study, while the National Health Research Authority provided the authorisation to conduct the study. A Material Transfer Agreement was also obtained from the National Health Research Authority to ship the plasma samples for testing and analysis at the Department of Epidemiology and Preventive Medicine, School of Public Health, Sackler Faculty of Medicine, Tel Aviv University, Tel Aviv, Israel.

## Results

### Exploring demographic differences between *S*. *flexneri* 2a and *S*. *sonnei*

A total of 159 participants were included in this study, among whom 53% were female and 37% were HIV-exposed. Majority of the mothers were above 24 years, and approximately 6% (10/160) of the infants were stunted according to the World Health Organisation (WHO) standards i.e. children are defined as stunted if their height-for-age is more than two standard deviations below the WHO Child Growth Standards median Table 1.

**Table 1:** Characteristics of the study population.

Characteristics	N (%)
**Gender**	
Female	84 (53.42)
Male	75 (46.58)
**HIV exposure**	
exposed	60 (37.27)
not exposed	101 (62.73)
**Diarrhoea Post Vaccination (n = 72)**	
No	68 (94.44)
Yes	4 (5.56)
**Mother’s age**	
<18	10 (6.21)
18–24	73 (45.34)
24>	78 (48.45)
**Wasted**	
No	91 (56.52)
Yes	70 (43.48)
**Stunted (n = 160)**	
No	150(93.8)
Yes	10(6.3)

### Kinetics of specific IgG antibodies to Shigella LPS

**Fig 2:**
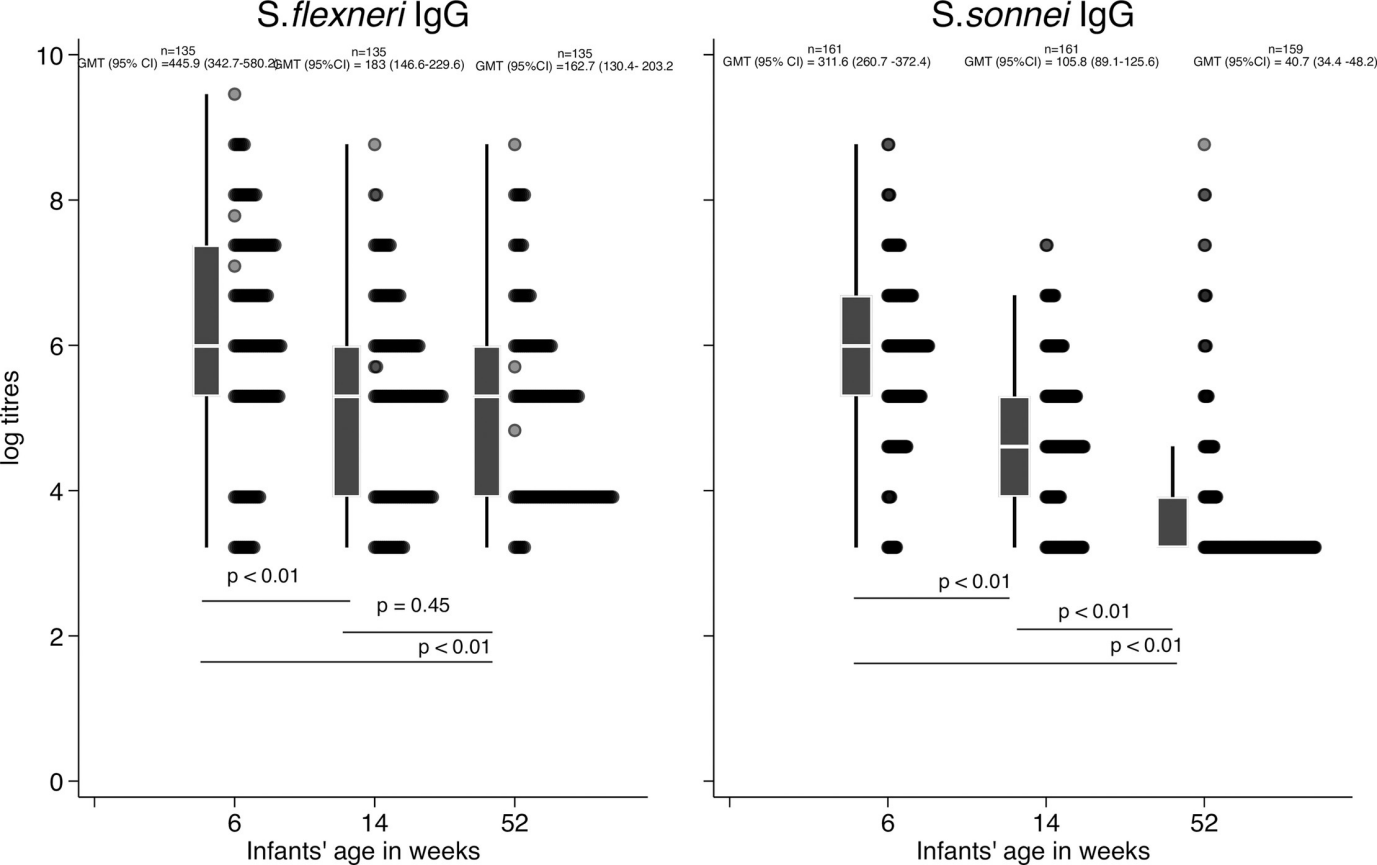
Kinetics of infant *Shigella* LPS specific IgG antibody titres form 6 to 52 weeks for *S*. *flexneri* 2a Panel A. Similarly, kinetics of *S*. *sonnei* Panel B. In both panels, transplacentally transferred maternal antibodies to *S*. *flexneri* 2a Panel A and *S*. *sonnei* Panel B were highest at 6 weeks and decreased by 52 weeks. The decrease is statistically significant between 6 and 14 and not between 14 and 52 weeks for antibodies to *S*. *flexneri* 2a LPS. A similar trend was observed for S. sonnei LPS IgG antibodies with the decrease when compared to 6 weeks titres statistically significant at all other time points.

At 6 weeks infant age, we found that the IgG GMT against *S*. *sonnei* was 311 (95% CI: 259–372; N = 159) whereas for *S*. *flexneri* 2a the IgG GMT was 446 (95% CI: 343–580; N = 135) (Fig 2). By 14 weeks lower IgG GMT were observed for both *S*. *sonnei* and *S*. *flexneri* 2a 104 (95% CI: 88–124) and 183 (95% CI: 147–230), implying a decrease of transplacental maternal IgG and this decreasing trend continued to 52 weeks of age at which IgG antibody titres against S. *sonnei* and S. *flexneri* 2a were 41 (95%CI: 34–48) and 163 (95%CI: 130–203) respectively.

**Fig 3:**
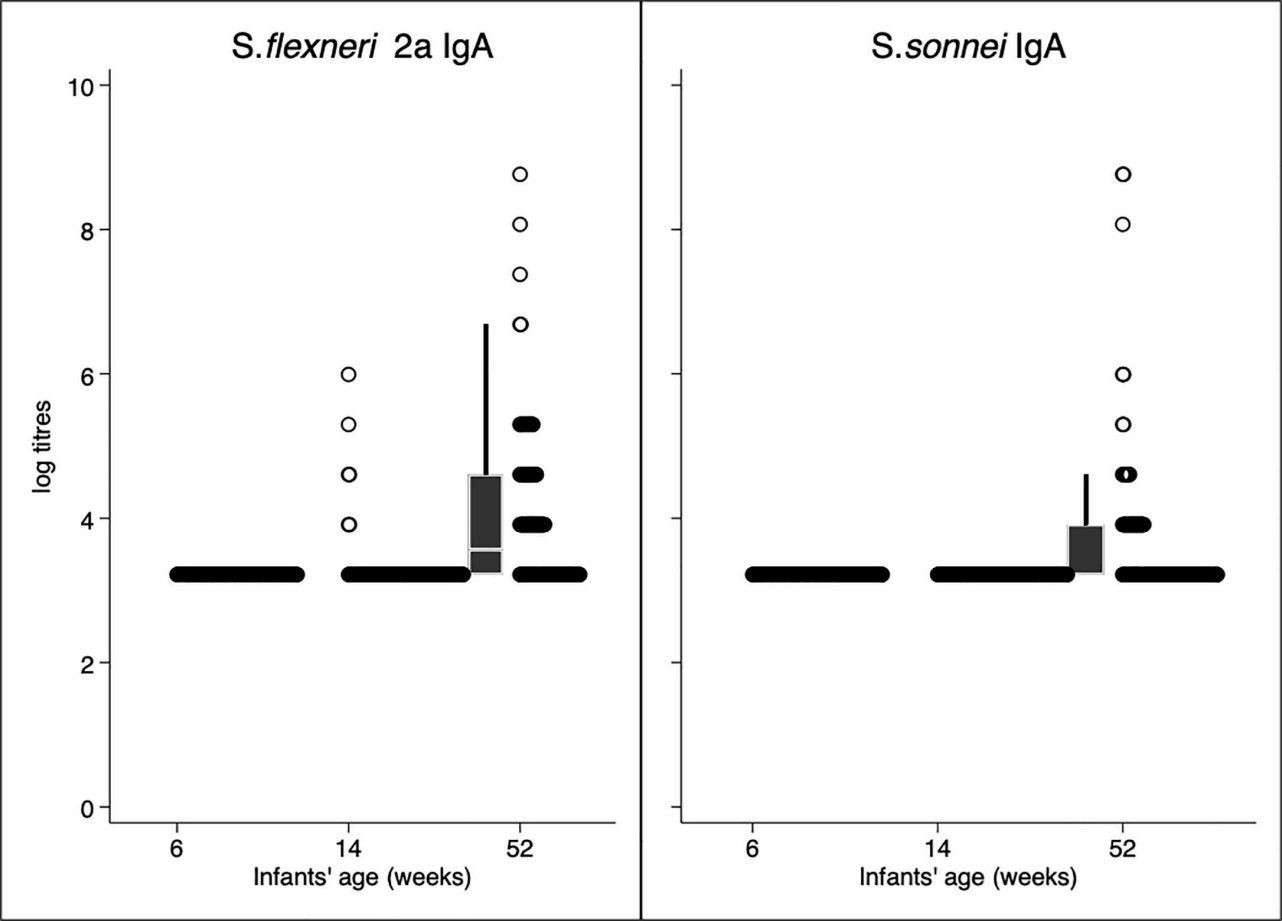
Kinetics of infant *Shigella* LPS specific IgA antibody titres to *S*. *flexneri* 2a in Panel A and kinetics of infant *Shigella* LPS specific IgA antibody titres to *S*. *sonnei* in Panel B. In both Panels, infant *Shigella* LPS specific IgA antibody titres to *S*. *flexneri* 2a and to *S*. *sonnei* were undetectable in most individuals with a few showing an elevation by 14 weeks and a few more by 52 weeks.

IgA GMT (95% CI) to *S*. *flexneri* 2a were <25 at 6 and 14 weeks and 52 (44–62) at 52 weeks. IgA GMT to *S*. *sonnei* were also <25 at 6 and week 14 weeks and 36 (31–41) by 52 weeks. In both instances, between 6 and 14 weeks IgA antibody titres were generally low with an observed rise between 14 and 52 weeks, indicative of potential exposure to *Shigella* in a period when infants are able to develop their own IgA Fig 3.

### Exploring demographic differences between *S*. *flexneri* 2a and *S*. *sonnei*

We also investigated differences in demographic characteristics between infants with increased IgG antibody titres between 52 and 14 weeks, for both *S*. *flexneri 2a* and *S*. *sonnei* and found no significant differences (Table 2).

**Table 2:** Differences between children with a significant 4-fold rise in titre to *S*. *flexneri 2a* LPS and *S*. *sonnei* LPS by demographic characteristics.

Characteristics	n(%) tested for S. *flexneri* at 52 and 14 weeks	*S*. *flexneri* 2a IgG rise (52 weeks vs 14 weeks) n = 135	p-value	n(%) tested for S. *sonnei* at 52 and 14 weeks	*S*. *sonnei* IgG rise (52 weeks vs 14 weeks) n = 159	p-value
**Gender**						
Female	72(53.3)	24 (33.3)	0.69	85(53.5)	14 (16.5)	0.19
Male	63(46.7)	23 (36.5)		74(46.5)	7 (9.5)	
**HIV exposure**						
Exposed	48(35.6)	18 (33.3)	0.63	60(37.7)	7 (11.7)	0.66
Unexposed	87(64.4)	29 (37.5)		99(62.3)	14 (14.1)	
**Mothers’ age**						
<18	6(4.4)	2 (33.3)	0.4	10(6.3)	1 (10)	0.94
18–24	64(47.4)	26 (40.6)		72(45.3)	10 (13.9)	
24>	65(48.1)	19 (29.2)		77(48.4)	10 (13)	
**Stunted** ^**2**^						
No	125(94.7)	44 (35.2)	1 ^1^	147(94.2)	19 (12.9)	0.6 ^1^
Yes	7(5.3)	2 (28.6)		9(5.8)	0 (0)	

^**1**^ **Fisher’s exact test.**

^**2**^ **n = 132 for *S*. *flexneri* and 159 for *S*. *sonnei*.**

### IgG anti-*S*. *sonnei* and *S*. *flexneri* 2a LPS response

We observed a similar trend in the decline over time of *S*. *flexneri* 2a and to *S*. *sonnei*-specific GMT with recorded higher IgG GMT between 6 to 14 weeks and the lowest at 52 weeks (Table 3). Likewise, the geometric mean fold change in titre (95%CI) between 14 to 52 weeks when compared to 6 to 14 weeks titre was 0.9; 95% CI: 0.7–1.2 for *S*. *flexneri* 2a and 0.4; 95% CI: 0.30–0.5 to *S*. *sonnei* respectively (Table 3). Whereas at 52 weeks, the geometric mean fold change in titres when compared to the titre at 6 weeks was 0.4; 95% CI: 0.3–0.5 to *S*. *flexneri* 2a and 0.1; 95% CI: 0.1–0.2 to *S*. *sonnei* (Table 3).

**Table 3:** Serum IgG anti-*S*. *flexneri* 2a or *S*. *sonnei* LPS response.

IgG:								
Period	# of infants		Geometric mean titre (95%CI) ^1^		Geometric mean fold change in titre (95%CI)		Seroconversion, n(%); 95%CI	
	*S*. *flexneri*	*S*. *sonnei*	*S*. *flexneri*	*S*. *sonnei*	*S*. *flexneri*	*S*. *sonnei*	*S*. *flexneri*	*S*. *sonnei*
Week 6–14	135	159	446 (343–580)	311 (259–372)	0.4 (0.3–0.5)	0.3 (0.3–0.4)	7 (5.2); (2.1–10.4)	5 (3.1); (1.0–7.1)
Week 14–52	135	159	183 (147–230)	104 (88–124)	0.9 (0.7–1.2)	0.4 (0.30–0.5)	36 (26.7); (19.4–35.0)	12 (7.5); (4.0–12.8)
Week 6–52	135	159	163 (130–203)	41 (34–48)	0.4 (0.3–0.5)	0.1(0.1–0.2)	21 (15.6); (9.9–22.8)	5 (3.1); (1.0–7.2)

We found that 5% infants showed a 4-fold rise in titre to *S*. *flexneri* 2a between 6 and 14 weeks, 27% between 14 and 52 weeks and finally 16% between 6–52 weeks (Table 3). Also, 3% infants showed a 4-fold rise in IgG titre to *S*. *sonnei* LPS between 6 and 14 weeks and 8% between 14 and 52 weeks respectively (Table 3).

IgA GMT between 6 to 14 weeks were lowest rising at 52 weeks (Table 4). Similarly, there was no observed response in the geometric mean fold change in titre (95%CI) between 6 and 14 weeks with only a minimal rise at 52 to *S*. *flexneri* 2a (2.1; 95% CI: 1.8–2.5) and to *S*. *sonnei* (1.4; 95% CI: 1.2–1.6) at 52 weeks (Table 4).

**Table 4:** Serum IgA anti-S. *flexneri* 2a or S. *sonnei* LPS response.

IgA:								
Period	# of infants		Geometric mean titre (95%CI) ^1^		Geometric mean fold change in titre (95%CI)		Seroconversion, n(%); 95%CI	
	*S*. *flexneri*	*S*. *sonnei*	*S*. *flexneri*	*S*. *sonnei*	*S*. *flexneri*	*S*. *sonnei*	*S*. *flexneri*	*S*. *sonnei*
Week 6–14	134	156	<25	<25	NR	NR	4* (3.0); (1.2–7.4)	NR
Week 14–52	134	156	<25	<25	NR	NR	37 (27.6); (20.7–35.7)	16 (10.3); (6.4–16.0)
Week 6–52	134	156	52 (44–62)	36 (31–41)	2.1 (1.8–2.5)	1.4 (1.2–1.6)	39 (29.1); (22.1–37.3)	16 (10.3); (6.4–16.0)

^1^GMT is reported at week 6, 14 and 52 respectively.

*4 respective titers on week 14 are: 200,100,100,400.

Approximately 3% individuals showed 4-fold rise in IgA titres to *S*. *flexneri* 2a between 6 and 14 weeks, 28% between 14 and 52 weeks and finally 29% between 6–52 weeks (Table 4). Also, 10% infants showed a 4-fold rise in IgA titres to *S*. *sonnei* LPS between 14 and 52 weeks and between 6 and 52 weeks respectively (Table 4).

### IgG and IgA antibody profile among infants

**Fig 4:**
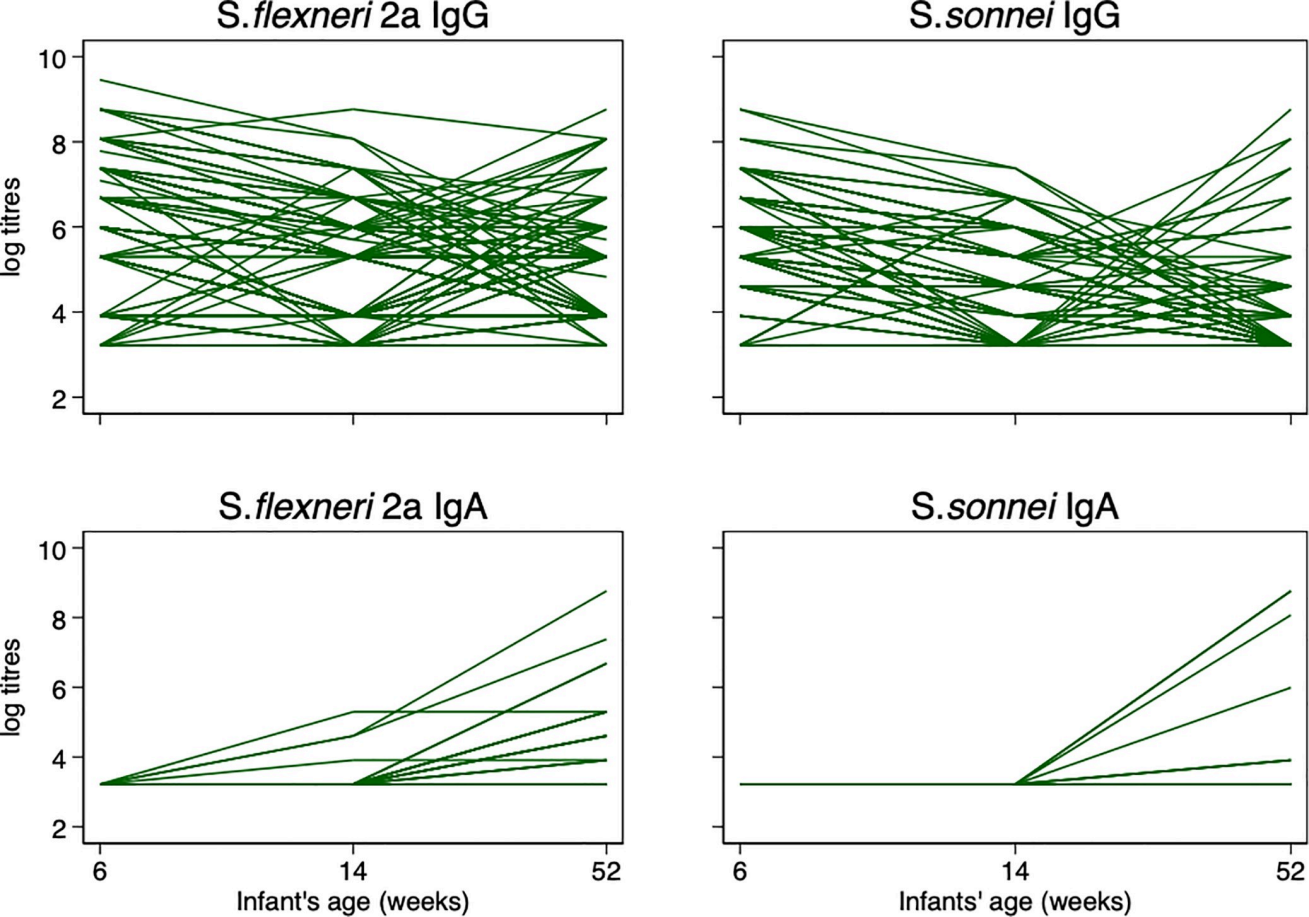
The individual IgG and IgA response profile among infants from 6 to 52 weeks.

When individual IgG antibody titres were further analysed, we found that *S*. *flexneri* 2a IgG GMT kinetics from 6 to 52 weeks infant age generally started out with a high GMT at 6 weeks, decreased at 14 weeks with a gradual increase from 14 weeks through 52 weeks (Fig 4). Also, a similar trend was noted in *S*. *sonnei* (Fig 4).

In both *S*. *flexneri* 2a and *S*. *sonnei*, IgA GMTs were very low at 6 weeks and increased markedly from 14 weeks onwards (Fig 4).

## Discussion

We carried out a sero-epidemiologic study of *S*. *flexneri* 2a and *S*. *sonnei* specific antibodies in the first year of life in Zambian infants and here report on maternal antibody transfer, decay and titre kinetics at the three time points for which we had available samples to test. Furthermore, our results are consistent with early exposure to Shigella we have as indicated by the 4-fold rise in IgG and IgA antibodies to *S*. *flexneri* 2a and *S*. *sonnei* LPS following the decrease in IgG transferred maternal antibodies.

Overall, our results indicate that between 6–14 weeks infant age IgG antibodies to both *S*. *flexneri* 2a and *S*. *sonnei* are highest and suggestive of transplacental transfer as has been demonstrated in reports from Israel [17] and Brazil [30]. Higher neonatal IgG titres have equally been reported for other pathogens like influenza virus [31], measles virus [32], and *Bordetella pertussis* as evidence of maternal transfer [33]. Similar work in Malawian children attributed reduced susceptibility to non-typhoidal *Salmonella* to transplacentally acquired antibodies [34, 35]. We thus infer that infants in our cohort were born from mothers with high levels of *Shigella* IgG anti-LPS antibodies passively acquiring these antibodies through trans placental transfer. It has been shown that serum IgG antibodies to *Shigella* LPS correlate with protection and this might explain the lowest incidence rates of shigellosis in the first year of life in both LMICs and high income countries endemic for shigellosis [15, 36].

Despite this assumption of maternal transfer in our cohort, our results must be interpreted with caution as we used left over plasma samples and were unable to test sera from the mothers to demonstrate correlations as shown elsewhere [33]. Further, due to limited clinical data, we could not analyse for factors that might influence efficient transfer of antibodies like maternal antibody concertation [33], pregnancy related complications (e.g. diabetes) [37] and gestation age [30].

We also observed a notable decrease of the transferred antibodies to both *S*. *flexneri* 2a and *S*. *sonnei* by 14 weeks, suggesting maternal antibody reduction. We thus postulated a gradual increase in infant immune competence. In fact, we began to see rises in IgG and IgA to both *S*. *flexneri* 2a and *S*. *sonnei* between 14–52 week time point suggesting that infants began to mount their own individual responses possibly in response to newly acquired infection. While we could not associate these observations to confirmed clinical disease, we postulate that this might indeed be the age around which supplementary feeding gets introduced, and infants begin to get exposed to potentially contaminated feeds [38] and to other modes of fecal-oral transmission (person-to-person, fomite and fly-borne).

Our results also showed that there were more infants responding to *S*. *flexneri* 2a compared to *S*. *sonnei* when data was analysed by group. Previous data show that generally *S*. *flexneri* 2a is much more common in LMICs [5] and data from Zambia seem to support this finding as *S*. *flexneri* was recently reported to be more common compared to other serotypes [39]. Higher prevalence of *flexneri* 2a in Zambia supports our observed drop in titres against *S*. *sonnei* by 52 weeks when compared to those of *S*. *flexneri* 2a. Additionally, the moderate drop in *S*. *flexneri* 2a antibodies between 14 and 52 weeks could mean exposure in some infants after maternal antibody deterioration. These findings are further strengthened by the notable percent of infants showing a significant rise in both IgG and IgA titres to both *S*. *flexneri* 2a and *S*. *sonnei* by 52 weeks. Furthermore, simultaneous fold-increase analysis showed that there were more infants with a four-fold rise in antibody titres at 52 weeks to *S*. *flexneri* 2a compared to *S*. *sonnei*. Nonetheless, it remains clear that *S*. *sonnei* infections are present and future *Shigella* vaccines should confer protection against these both strains.

It appears that a potential timing of vaccination against shigellosis in infants in Zambia would be after 14 and before 52 weeks of age because: i) the possible interference with maternally acquired antibodies decreases after 14 weeks; ii) infants are still within the age-eligible period for visits for routine immunisations for other vaccines such as those against pneumonia, diphtheria, pertussis etc. and iii) the risk of natural exposure to infection begins to rise as most mothers start to introduce supplementary feeds and as a result of other modes of fecal-oral transmission (person-to-person, fomite and fly-borne).

Indeed other studies investigating maternal antibody longevity have likewise reported a drop in maternal antibodies around the same period [18, 40]. However, while it is postulated that administration of vaccines close to when maternal antibodies decrease would be very attractive, it is not guaranteed as some evidence has shown that residual concentration of maternal placentally transferred antigen-specific antibodies at the time of immunisation potentially inhibit the immune response to infant vaccination against some antigens [41]. It remains an important research question as to whether such residual maternal antibodies will actually interfere with vaccine uptake or functionality of induced vaccine responses in LMIC infants. Another important factor for future decisions on the timing of administration of the first dose of vaccine will be the immunogenicity of the candidate vaccine so early in infancy.

Our study has several strengths as well as limitations. Notable strengths include first, the measurement of IgG and IgA antibodies to *Shigella* LPS which makes it possible to assess the individual infant exposure to both *S*. *flexneri 2a* and *S*. *sonnei* at the various points in a longitudinal cohort of infants; to our knowledge, this is the first such longitudinal observation in African infants. While it appears that a potential timing of vaccination would be after 14 and before 52 weeks of age to ensure early infant protection against shigellosis, a more comprehensive sero-epidemiological study together with the month on month incidence of shigellosis will help indicate a narrower and optimal age for vaccination.

Limitations include the inability to assess the associations between infant titres perceived to be acquired by placental transfer and maternal serum titre levels. The study also had insufficient data on clinical disease as the passive diarrhoea surveillance captured only a few cases that self-reported to the facility; this would miss all the subclinical infections or even those managed at home, and yet such infections still contribute to the evolution of immunity against disease in the infant.

Nonetheless, these data do contribute to global knowledge on shigellosis in endemic settings and certainly demonstrate early natural exposure in to *Shigella* in infants. Here we affirm the need for a preventive vaccine and also propose an optimal window for delivery of the vaccine.

## Conclusion

*Shigella* exposure is common in LMICs and maternal IgG is readily transferred across the placenta, decreasing by 14 weeks of age in the majority of infants. Early natural exposure to *Shigella* thereafter is associated with significant rises in specific antibodies to *S*. *flexneri* 2a and *S*. *sonnei* LPS. A vaccine against shigellosis is needed and is desirable to target both *S*. *flexneri 2a* and *S*. *sonnei* serotypes within a multivalent formulation. A potential timing of vaccination would be after 14 and before 52 weeks of age to ensure early infant protection against shigellosis.

## Recommendations

Appropriate monitoring and prevention strategies can be targeted to such vulnerable population with a marginally primed immune system.

## References

[1] Levine MM, Nasrin D, Acácio S, Bassat Q, Powell H, Tennant SM, et al. Diarrhoeal disease and subsequent risk of death in infants and children residing in low-income and middle-income countries: analysis of the GEMS case-control study and 12-month GEMS-1A follow-on study. Lancet Glob Heal. 2020;8: e204–e214. 10.1016/S2214-109X(19)30541-8 31864916 PMC7025325

[2] Vos T, Allen C, Arora M, Barber RM, Brown A, Carter A, et al. Global burden of childhood pneumonia and diarrhoea. Lancet. 2013;388: 1405–1416. 10.1016/S0140-6736(13)60222-6 23582727 PMC7159282

[3] Vos T, Allen C, Arora M, Barber RM, Brown A, Carter A, et al. Global, regional, and national incidence, prevalence, and years lived with disability for 310 diseases and injuries, 1990–2015: a systematic analysis for the Global Burden of Disease Study 2015. Lancet. 2016;388: 1545–1602. 10.1016/S0140-6736(16)31678-6 27733282 PMC5055577

[4] Kotloff KL, Platts-Mills JA, Nasrin D, Roose A, Blackwelder WC, Levine MM. Global burden of diarrheal diseases among children in developing countries: Incidence, etiology, and insights from new molecular diagnostic techniques. Vaccine. Netherlands; 2017;35: 6783–6789. 10.1016/j.vaccine.2017.07.036 28765005

[5] Livio S, Strockbine NA, Panchalingam S, Tennant SM, Barry EM, Marohn ME, et al. Shigella isolates from the global enteric multicenter study inform vaccine development. Clin Infect Dis. 2014;59: 933–941. 10.1093/cid/ciu468 24958238 PMC4166982

[6] Chisenga CC, Bosomprah S, Makabilo Laban N, Mwila- Kazimbaya K, Mwaba J, Simuyandi M, et al. vAetiology of Diarrhoea in Children Under Five in Zambia Detected Using Luminex xTAG Gastrointestinal Pathogen Panel. Pediatr Infect Dis Open Access. 2018;03: 1–6. 10.21767/2573-0282.100064

[7] Scallan E, Hoekstra RM, Angulo FJ, Tauxe R V., Widdowson MA, Roy SL, et al. Foodborne illness acquired in the United States-Major pathogens. Emerg Infect Dis. 2011;17: 7–15. 10.3201/eid1701.p11101 21192848 PMC3375761

[8] Thompson CN, Duy PT, Baker S. The Rising Dominance of Shigella sonnei: An Intercontinental Shift in the Etiology of Bacillary Dysentery. PLoS Negl Trop Dis. Public Library of Science; 2015;9: e0003708–e0003708. 10.1371/journal.pntd.0003708 26068698 PMC4466244

[9] Darton TC, Tuyen HT, The HC, Newton PN, Dance DAB, Phetsouvanh R, et al. Azithromycin Resistance in Shigella spp. in Southeast Asia. Antimicrob Agents Chemother. American Society for Microbiology; 2018;62: e01748–17. 10.1128/AAC.01748-17 29378707 PMC5913960

[10] Chung The H, Rabaa MA, Pham Thanh D, De Lappe N, Cormican M, Valcanis M, et al. South Asia as a Reservoir for the Global Spread of Ciprofloxacin-Resistant Shigella sonnei: A Cross-Sectional Study. PLoS Med. 2016;13. 10.1371/journal.pmed.1002055 27483136 PMC4970813

[11] Mani S, Wierzba T, Walker RI. Status of vaccine research and development for Shigella. Vaccine. Elsevier Ltd; 2016;34: 2887–2894. 10.1016/j.vaccine.2016.02.075 26979135

[12] Lerman Y, Yavzori M, Ambar R, Sechter I, Wiener M, Cohen D. Epidemic spread of Shigella sonnei shigellosis and evidence for development of immunity among children attending day-care centers in a communal settlement (Kibbutz). J Clin Microbiol. American Society for Microbiology; 1994;32: 1092–4. Available: http://www.ncbi.nlm.nih.gov/pubmed/7913096 10.1128/JCM.32.4.1092-1094.1994 7913096 PMC267193

[13] Ferreccio C, Prado V, Ojeda A, Cayyazo M, Abrego P, Guers L, et al. Epidemiologic Patterns of Acute Diarrhea and Endemic Shigella Infections in Children in a Poor Periurban Setting in Santiago, Chile. Am J Epidemiol. 1991;134: 614–627. Available: 10.1093/oxfordjournals.aje.a116134 1951266

[14] COHEN D, BASSAL R, GOREN S, ROUACH T, TARAN D, SCHEMBERG B, et al. Recent trends in the epidemiology of shigellosis in Israel. Epidemiol Infect. 2014/02/20. Cambridge University Press; 2014;142: 2583–2594. 10.1017/S0950268814000260 24559503 PMC9151250

[15] Kotloff KL, Nataro JP, Blackwelder WC, Nasrin D, Farag TH, Panchalingam S, et al. Burden and aetiology of diarrhoeal disease in infants and young children in developing countries (the Global Enteric Multicenter Study, GEMS): a prospective, case-control study. Lancet. Elsevier; 2013;382: 209–222. 10.1016/S0140-6736(13)60844-2 23680352

[16] Robbins JB, Chu C, Schneerson R. Hypothesis for Vaccine Development: Protective Immunity to Enteric Diseases Caused by Nontyphoidal Salmonellae and Shigellae May Be Conferred by Serum IgG Antibodies to the O-Specific Polysaccharide of Their Lipopolysaccharides. Clin Infect Dis. 1992;15: 346–361. Available: 10.1093/clinids/15.2.346 1381621

[17] Passwell JH, Freier S, Shor R, Farzam N, Block C, Lison M, et al. Shigella lipopolysaccharide antibodies in pediatric populations. Pediatr Infect Dis J. United States; 1995;14: 859–865. 10.1097/00006454-199510000-00008 8584312

[18] Thompson CN, Tu LTP, Anders KL, Hieu NT, Vi LL, Chau NVV, et al. The transfer and decay of maternal antibody against Shigella sonnei in a longitudinal cohort of Vietnamese infants. Vaccine. Elsevier Science; 2016;34: 783–790. 10.1016/j.vaccine.2015.12.047 26742945 PMC4742520

[19] Cohen D, Green MS, Block C, Rouach T, Ofek I. Serum Antibodies to Lipopolysaccharide and Natural Immunity to Shigellosis in an Israeli Military Population. J Infect Dis. Oxford University Press; 1988;157: 1068–1071. Available: http://www.jstor.org/stable/30135717 10.1093/infdis/157.5.1068 3283258

[20] Cohen D, Green MS, Block C, Slepon R, Ofek I. Prospective study of the association between serum antibodies to lipopolysaccharide O antigen and the attack rate of shigellosis. J Clin Microbiol. American Society for Microbiology; 1991;29: 386–9. Available: http://www.ncbi.nlm.nih.gov/pubmed/1706731 10.1128/JCM.29.2.386-389.1991 1706731 PMC269772

[21] Cohen D, Ashkenazi S, Green MS, Gdalevich M, Robin G, Slepon R, et al. Double-blind vaccine-controlled randomised efficacy trial of an investigational Shigella sonnei conjugate vaccine in young adults. Lancet. Elsevier; 2018;349: 155–159. 10.1016/S0140-6736(96)06255-1 9111538

[22] Passwell JH, Ashkenzi S, Banet-Levi Y, Ramon-Saraf R, Farzam N, Lerner-Geva L, et al. Age-related efficacy of Shigella O-specific polysaccharide conjugates in 1–4-year-old Israeli children. Vaccine. 2010;28: 2231–2235. 10.1016/j.vaccine.2009.12.050 20056180 PMC6503522

[23] Cohen D, Atsmon J, Artaud C, Meron-Sudai S, Gougeon ML, Bialik A, et al. Safety and immunogenicity of a synthetic carbohydrate conjugate vaccine against Shigella flexneri 2a in healthy adult volunteers: a phase 1, dose-escalating, single-blind, randomised, placebo-controlled study. Lancet Infect Dis. 2021 4;21(4):546–558. 10.1016/S1473-3099(20)30488-6 Epub 2020 Nov 10. . 33186516

[24] Riddle MS, Kaminski RW, Di Paolo C, Porter CK, Gutierrez RL, Clarkson KA, et al. Safety and Immunogenicity of a Candidate Bioconjugate Vaccine against Shigella flexneri 2a Administered to Healthy Adults: a Single-Blind, Randomized Phase I Study. Pasetti MF, editor. Clin Vaccine Immunol. 1752 N St., N.W., Washington, DC: American Society for Microbiology; 2016;23: 908–917. 10.1128/CVI.00224-16 27581434 PMC5139601

[25] Gerke C, Colucci AM, Giannelli C, Sanzone S, Vitali CG, Sollai L, et al. Production of a Shigella sonnei Vaccine Based on Generalized Modules for Membrane Antigens (GMMA), 1790GAHB. PLoS One. Public Library of Science; 2015;10: e0134478. Available: 10.1371/journal.pone.0134478 26248044 PMC4527750

[26] Obiero CW, Ndiaye AGW, Sciré AS, Kaunyangi BM, Marchetti E, Gone AM, et al. A Phase 2a Randomized Study to Evaluate the Safety and Immunogenicity of the 1790GAHB Generalized Modules for Membrane Antigen Vaccine against Shigella sonnei Administered Intramuscularly to Adults from a Shigellosis-Endemic Country. Frontiers in Immunology. 2017. p. 1884. 10.3389/fimmu.2017.01884 29375556 PMC5763125

[27] Kawsar R. Talaat, Cristina Alaimo, A. Lou Bourgeois, Robert W. Kaminski, Anita Dreyer, Chad K. Porter, et al. Flexyn2a, a candidate bioconjugate vaccine against Shigella flexneri 2a induces protective immune responses in a controlled human infection model. 9th International Conference on Vaccines for Enteric Diseases. 2017.

[28] Chilengi R, Simuyandi M, Beach L, Mwila K, Becker-Dreps S, Emperador DM, et al. Association of Maternal Immunity with Rotavirus Vaccine Immunogenicity in Zambian Infants. PLoS One. Public Library of Science; 2016;11: e0150100. Available: 10.1371/journal.pone.0150100 26974432 PMC4790930

[29] Cohen D, Block C, Green MS, Lowell G, Ofek I. Immunoglobulin M, A, and G antibody response to lipopolysaccharide O antigen in symptomatic and asymptomatic Shigella infections. J Clin Microbiol. 1989;27: 162–167. 10.1128/JCM.27.1.162-167.1989 2463995 PMC267253

[30] Costa-Carvalho BT, Vieria HM, Dimantas RB, Arslanian C, Naspitz CK, Solé D, et al. Transfer of IgG subclasses across placenta in term and preterm newborns. Brazilian J Med Biol Res = Rev Bras Pesqui medicas e Biol. Departamento de Pediatria, Universidade de São Paulo, Brasil.; 1996;29: 201–204. Available: http://europepmc.org/abstract/MED/8731349 8731349

[31] Vilajeliu A, Goncé A, López M, Costa J, Rocamora L, Ríos J, et al. Combined tetanus-diphtheria and pertussis vaccine during pregnancy: transfer of maternal pertussis antibodies to the newborn. Vaccine. 2015;33: 1056–1062. 10.1016/j.vaccine.2014.12.062 25573035

[32] Mäntyjärvi R, Hirvonen T, Toivanen P. Maternal antibodies in human neonatal sera. Immunology. 1970;18: 449–451. 5442221 PMC1455561

[33] Gonçalves G, Cutts FT, Hills M, Rebelo-Andrade H, Trigo FA, Barros H. Transplacental transfer of measles and total IgG. Epidemiol Infect. 1999;122: 273–279. 10.1017/s0950268899002046 10355792 PMC2809616

[34] MacLennan CA, Gondwe EN, Msefula CL, Kingsley RA, Thomson NR, White SA, et al. The neglected role of antibody in protection against bacteremia caused by nontyphoidal strains of Salmonella in African children. J Clin Invest. The American Society for Clinical Investigation; 2008;118: 1553–1562. 10.1172/JCI33998 18357343 PMC2268878

[35] Nyirenda TS, Gilchrist JJ, Feasey NA, Glennie SJ, Bar-Zeev N, Gordon MA, et al. Sequential Acquisition of T Cells and Antibodies to Nontyphoidal Salmonella in Malawian Children. J Infect Dis. 2014;210: 56–64. 10.1093/infdis/jiu045 24443544 PMC4054899

[36] Cohen D, Muhsen K. Vaccines for enteric diseases. Hum Vaccin Immunother. Taylor & Francis; 2019;15: 1205–1214. 10.1080/21645515.2019.1611200 31291174 PMC6663139

[37] França EL, Calderon IDMP, Vieira EL, Morceli G, Honorio-França AC. Transfer of maternal immunity to newborns of diabetic mothers. Clin Dev Immunol. 2012;2012. 10.1155/2012/928187 22991568 PMC3444004

[38] Greenland K, Chipungu J, Curtis V, Schmidt W-P, Siwale Z, Mudenda M, et al. Multiple behaviour change intervention for diarrhoea control in Lusaka, Zambia: a cluster randomised trial. Lancet Glob Heal. 2016;4: e966–e977. 10.1016/S2214-109X(16)30262-5 27855872

[39] Chiyangi H, Muma JB, Malama S, Manyahi J, Abade A, Kwenda G, et al. Identification and antimicrobial resistance patterns of bacterial enteropathogens from children aged 0–59 months at the University Teaching Hospital, Lusaka, Zambia: A prospective cross sectional study. BMC Infect Dis. BMC Infectious Diseases; 2017;17: 1–9. 10.1186/s12879-016-2122-x 28152988 PMC5290660

[40] De Alwis R, Tu LTP, Quynh NLT, Thompson CN, Anders KL, Van Thuy NT, et al. The Role of Maternally Acquired Antibody in Providing Protective Immunity Against Nontyphoidal Salmonella in Urban Vietnamese Infants: A Birth Cohort Study. J Infect Dis. 2019;219: 295–304. 10.1093/infdis/jiy501 30321351 PMC6306017

[41] Voysey M, Kelly DF, Fanshawe TR, Sadarangani M, O’Brien KL, Perera R, et al. The Influence of Maternally Derived Antibody and Infant Age at Vaccination on Infant Vaccine Responses: An Individual Participant Meta-analysis. JAMA Pediatr. American Medical Association; 2017;171: 637–646. 10.1001/jamapediatrics.2017.0638 28505244 PMC5710349

